# Ventricular Arrhythmias in a Patient With Propionic Acidemia

**DOI:** 10.7759/cureus.28966

**Published:** 2022-09-09

**Authors:** Anthony A Della Rossa, Priyadarshini M Dixit, Ruchit Shah, Stephanie Hang, Jacky Duong

**Affiliations:** 1 Internal Medicine, Saint Joseph Mercy Oakland, Pontiac, USA; 2 Cardiology, Saint Joseph Mercy Oakland, Pontiac, USA

**Keywords:** cardiology devices, ekg abnormalities, ekg, qt prolongation, aicd, cardiac electrophysiology

## Abstract

Propionic acidemia (PA) is a metabolic disorder that involves a defective copy of propionyl-CoA carboxylase (PCC). It has previously been shown that there is an association between QT-prolongation in propionic acidemia. The patient seen in this case is a male in his early twenties with known PA who was found unconscious on initial presentation due to cardiac arrest with a downtime of twenty minutes. He was subsequently resuscitated and stabilized. The patient underwent placement of an automatic implantable cardioverter-defibrillator (AICD) nineteen days after the initial presentation.

## Introduction

Propionic Acidemia (PA) is an autosomal recessive disorder caused by a deficiency of the propionyl-CoA carboxylase (PCC) enzyme which is involved in converting propionyl-CoA to methylmalonyl-CoA [1]. In the neonatal period, patients with PA are seen to have decreased arousal, poor feeding, and progressive encephalopathy. Failure to diagnose PA in the early stages of life can lead to seizures or coma that can eventually result in death [2], Chapman et al. reported laboratory features associated with PA to include lactic acidosis, anion gap metabolic acidosis, ketonuria, hyperammonemia, and hypoglycemia. Patients with late-onset PA may be asymptomatic until they are under catabolic stress (usually involving fasting, illness, or surgery) and may present with vomiting, hypotonia, developmental delays, or cardiomyopathy. Some of the more common developments of cardiomyopathy involve QT-prolongation, hypertrophy, and dilated cardiomyopathy which predisposes patients to fatal cardiac arrhythmias [3]. It has been proposed that the cause is due to intracellular calcium mishandling leading to cardiac dysfunction and ventricular arrhythmias which were recently demonstrated in mouse models by Tamayo et al. [4]. 

## Case presentation

A 23-year-old male who was previously diagnosed with propionic acidemia in 2012 (on levocarnitine and nadolol) and cardiomegaly (first observed on a 2D echocardiogram in 2016) presented to the emergency department with cardiopulmonary arrest. He had witnessed a syncopal episode after which a family member performed CPR for ~10mins until emergency medical service (EMS) arrived. Upon the arrival of EMS, the initial rhythm showed ventricular fibrillation. Compressions were started, a bag-valve-mask was used for ventilation, pads were placed by EMS, and return of spontaneous circulation (ROSC) was achieved in the field after two rounds of defibrillation without the use of antiarrhythmics. Total downtime was estimated to be twenty minutes.

**Figure 1:**
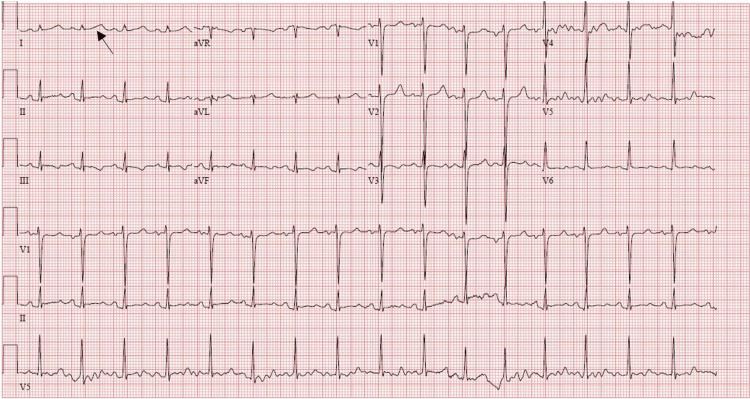
Electrocardiogram (EKG) demonstrating normal sinus rhythm T-wave inversions in leads III and aVF as well as a biphasic T-wave in lead V5. QTc noted to be 523ms.

Upon arrival at the emergency department, a repeat electrocardiogram (EKG) was done and showed a normal sinus rhythm, PR interval of 170ms, QRS duration of 84ms, a slightly prolonged QT, and normal axis deviation. T-wave inversion was noted in leads III and aVF as well as a biphasic T-wave in lead V5 (Figure 1). Vitals signs at presentation were unstable and showed tachycardia at 138/min, tachypneic at 45/min, BP at 137/84 mmHg, and SpO2 at 99% on room air. Physical examination was unremarkable except for dental fractures due to falling from standing height caused by the arrhythmia. Abnormal lab values on initial presentation included a serum blood urea nitrogen (BUN) of 4mg/dL, CO2 of 17mmol/L, glucose of 224, anion gap metabolic acidosis with an anion gap of 22mmol/L, HCO3- of 17mEq/L, and ammonia level of 128mg/L. Troponin peaked at 0.37 ng/ml. Sodium, potassium, chloride, and calcium levels were within normal range. Arterial blood gases showed acute hypercapnic respiratory failure (pH of 7.28, pCO2 of 51.5mmHg, pO2 of 518mmHg, HCO3- of 24mEq/L, and lactic acid of 7.5mmol/L) and were subsequently intubated for airway protection. CT head without contrast revealed no acute intracranial process. The patient was triaged to the intensive care unit (ICU) for further management.

A physical exam completed in the intensive care unit showed that the patient was sedated, ventilated, and occasionally withdrawing from pain. Neurological examination showed intact brainstem and reflexes. Cardiovascular examination revealed regular rate and rhythm with no murmurs or rubs. Breath sounds were equal bilateral with no rales, rhonchi, or wheezing and good air exchange. Bowel sounds were present and the abdomen was soft, and non-tender with no rebound or guarding. Given clinical presentation and examination findings, the patient met the criteria for targeted temperature management. Hypothermia protocol was initiated.

**Figure 2:**
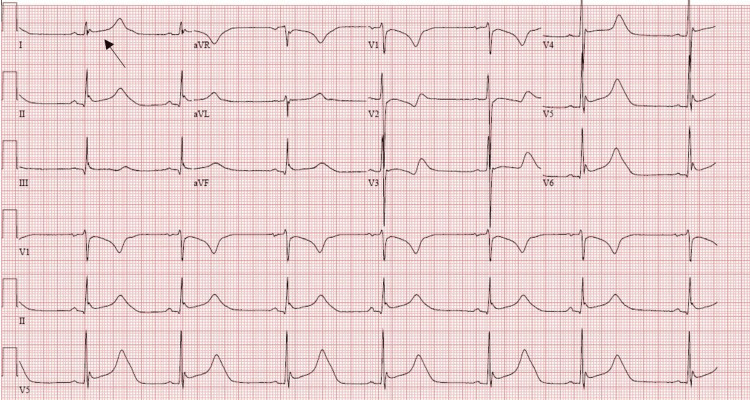
Sinus Bradycardia with prolonged QTc (627 ms), likely due to hypothermia protocol initiation.

Given the known history of PA, anion gap metabolic acidosis secondary to PA; the patient was started on 1g levocarnitine every six hours along with IV fluids with 5% dextrose (D5W) in normal saline, nadolol 60mg daily, and Keppra 500mg twice daily. The patient maintained sinus rhythm and was monitored without specific antiarrhythmic therapy. The target temperature (32-34 degrees Celsius) was reached. The re-warming phase was initiated. Twenty-four hours post-re-warming phase electroencephalogram (EEG) was done which did not demonstrate any findings suggestive of seizure activity/reduced waveform. EKG was ordered daily to monitor QTc interval which continued to remain prolonged despite medications (Figure 2). A transthoracic echocardiogram was obtained and showed a severely reduced ejection fraction of 30-35% with global hypokinesia, a grade II diastolic dysfunction, and mild pericardial effusion

As the patient had a good neurological response after the hypothermia protocol was completed, electrophysiology was consulted for recommendations on placement of an automated implantable defibrillator (AICD) as criteria were met for secondary prevention of cardiac arrest. Cardiac MRI was not completed at this time. The patient was transferred to a tertiary medical center for AICD placement. AICD placement has been shown to reduce or prevent the likelihood of additional future fatal arrhythmias [5].

## Discussion

PA has long been known to cause QT-prolongation [6] possibly due to the effects of toxic metabolites or the deprivation of essential substrates [7]. This in turn has been shown to lead to mitochondrial dysfunction, increased reactive oxygen species, and oxidative damage [4], Tamayo et al. While QT-prolongation can lead to cardiac arrhythmias such as torsades de pointes and ventricular fibrillation, new studies are seeking to understand the underlying cause which leads to these arrhythmias. One promising study is using mouse models with PA to show that intracellular calcium mishandling may play a role in ventricular arrhythmias [4], Tamayo, et al. The study used propionyl-CoA carboxylase subunit A (PCCA) -/- (A138T) knockout mice to mimic the characteristics of PA. What was seen was that these mice presented with diminished cell contractility and cardiac dysfunction associated with impairment in the sarcoplasmic reticulum Ca2+ load, lower systolic Ca2+ release, and decreased Ca2+ reuptake by sarcoplasmic Ca2+ ATPase (SERCA). The decrease in SERCA activity was shown to lead to an increase in diastolic Ca2+ release. This ultimately was shown to cause an increase in Ca2+ which could be the potential cause for induction of ventricular arrhythmias.

Induced pluripotent stem cells (iPSC) from patients with PA are currently being conducted on mutated mitochondrial copies of propionyl-CoA carboxylase subunit A & B (PCCA & PCCB respectively) [8]. Major causes of mortality involving cardiac alterations include dilated cardiomyopathy, long QT syndrome, and hypertrophy. PCCA iPCS-grown cardiomyocytes were shown to have reduced oxygen consumption, an increase in the accumulation of lipid droplets and residual bodies, and an increase in ribosomal formation. Additionally, endoplasmic reticulum stress along with calcium release has been tied to an increase in protein levels of glucose-regulated protein 75 (GRP75), glucose-regulated protein 78 (GRP78), homocysteine-inducible ER stress protein (HERP), mitofusin-2 (MFN2), and sigma-1 receptor (SIG-1R). 

Anesthetic complications arose when sedative agents proved to be ineffective in the placement of lines and monitors. Further investigations disclosed that common agents used as sedatives or for rapid sequence induction, particularly ones involved in the metabolization of propionic acids such as succinylcholine, neuromuscular blocking agents, and propofol were ineffective and can potentially exacerbate acidemia [9].

## Conclusions

This case goes over a young patient with PA who initially presented to us after experiencing ventricular arrhythmia. The pathophysiology of PA initially presents at or shortly after birth with signs of lethargy, vomiting, failure to thrive, hypotonia, and dehydration. Patients with PA are typically diagnosed at birth on expanded newborn screening tests and should be monitored closely by various specialties, most importantly cardiology and nutrition. Lifelong monitoring is required for the progression of cardiomyopathies and nutritional supplementation, which includes protein restrictions, formulas enriched with leucine, and foods free of isoleucine, methionine, threonine, and valine. As QT-prolongation progresses, ventricular arrhythmias may arise requiring a tertiary healthcare center. Preventative measures, requiring AICD placement, may be required to avoid ventricular arrhythmias which may lead to death as there is no definitive cure for PA.
